# Widely Targeted Metabolomic Analysis Reveals the Improvement in *Panax notoginseng* Triterpenoids Triggered by Arbuscular Mycorrhizal Fungi via UPLC–ESI–MS/MS

**DOI:** 10.3390/molecules29133235

**Published:** 2024-07-08

**Authors:** Xing-Kai Zhang, Yue Wu, Xian-Nv Long, Xiao-Xu You, Di Chen, Yue Bi, Sen He, Guan-Hua Cao

**Affiliations:** 1 School of Chinese Materia Medica, Yunnan University of Chinese Medicine, Kunming 650500, China; zxkaky@outlook.com (X.-K.Z.);; 2 Kunming Lancang-Mekong Regional R&D Central for the Development Utilization of Traditional Medicine Resources, Yunnan University of Chinese Medicine, Kunming 650500, China

**Keywords:** *Panax notoginseng*, arbuscular mycorrhizal fungi (AMF), widely targeted metabolomics, major notoginsenosides (MNs), rare notoginsenosides (RNs)

## Abstract

*Panax notoginseng* is a highly valued perennial medicinal herb in China and is widely used in clinical treatments. The main purpose of this study was to elucidate the changes in the composition of *P. notoginseng* saponins (PNSs), which are the main bioactive substances, triggered by arbuscular mycorrhizal fungi (AMF) via ultrahigh-performance liquid chromatography–electrospray ionization–tandem mass spectrometry (UPLC–ESI–MS/MS). A total of 202 putative terpenoid metabolites were detected, of which 150 triterpene glycosides were identified, accounting for 74.26% of the total. Correlation analysis, principal component analysis (PCA) and orthogonal partial least squares discriminant analysis (OPLS–DA) of the metabolites revealed that the samples treated with AMF (group Ce) could be clearly separated from the CK samples. In total, 49 differential terpene metabolites were identified between the Ce and CK groups, of which 38 and 11 metabolites were upregulated and downregulated, respectively, and most of the upregulated differentially abundant metabolites were mainly triterpene glycosides. The relative abundances of the two major notoginsenosides (MNs), ginsenosides Rd and Re, and 13 rare notoginsenosides (RNs), significantly increased. The differential saponins, especially RNs, were more easily clustered into one branch and had a high positive correlation. It could be concluded that the biosynthesis and accumulation of some RNs share the same pathways as those triggered by AMF. This study provides a new way to obtain more notoginsenoside resources, particularly RNs, and sheds new light on the scientization and rationalization of the use of AMF agents in the ecological planting of medicinal plants.

## 1. Introduction

*Panax notoginseng* (Burk.) F. H. Chen is a highly valued perennial medicinal herb of the genus *Panax*, family Araliaceae, and is widely planted in Yunnan Province, China. Notoginseng radix et rhizoma, the taproots of *P. notoginseng*, have been used as a tonic and hemostatic drugs for hundreds of years, in which time more than 200 active compounds have been isolated, including saponins, polysaccharides and flavonoids [1,2,3,4]. Evidence shows that *P. notoginseng* saponins (PNSs) are the main bioactive compounds and can be classified into two dammarane-type saponins, 20(S)-protopanaxatriol saponins (PTSs) and 20(S)-protopanaxadiol saponins (PDSs). PTSs contain notoginsenosides R1, R2 and R3 and ginsenosides Rg1, Rg2, Re, Rh1 and F1, whereas ginsenosides Rb1, Rb2, Rb3, Rc Rd, CK and F2 and notoginsenosides Fa, Fc Fe and D are PDSs [5,6]. In terms of the contents of notoginseng Radix et rhizoma, PNSs are usually divided into major notoginsenosides (MNs) and rare notoginsenosides (RNs). In many studies, ginsenosides Rb1, Rg1, Re and Rd, and notoginsenoside R1 were commonly classified as MNs that collectively constitute over 90% of PNSs [7]. The other saponins in *P. notoginseng* are grouped into RNs such as ginsenosides Rg2, Rg3, Rg4, Rg5, Rg6, Rh15 and Rh17, and notoginsenosides FP2, Fc, N and K [8,9].

Pharmacological studies have demonstrated that PNSs have powerful functions in terms of cardioprotective effects, protection against cerebrovascular injury, neuroprotective effects, antitumor activities, anti-inflammatory activities, hemostasis and anticoagulation [10,11,12,13]. Interestingly, most RNs exhibit greater biological activity [14,15,16].

However, the biosynthesis and accumulation of these notoginsenosides are influenced by numerous environmental and biological factors [17,18]. Owing to complicated pathways, the artificial synthesis of saponins is difficult. Therefore, the use of biological means to increase the accumulation of notoginsenosides, especially RNs, has become one of the focuses of attention.

The application of bacterial or fungal fertilizer is one of the most common means to increase yields, and the activity of these compounds has increased under modern agricultural management [19,20]. Arbuscular mycorrhizal fungi (AMF) are symbiotic endophytic fungi with a clear function, and they can form a mutualistic symbiosis with more than 80% of the roots of plant species in natural and agricultural systems [21,22]. Many studies have shown that the application of AMF fertilizers improved the growth and accumulation of secondary metabolites in medicinal plants, e.g., artemisinin of *Artemisia annua* L. [23,24], glycyrrhizin and liquiritin of *Glycyrrhiza uralensis* Fisch. [25,26] and aloe–emodin of *Polygonum cuspidatum* Sieb. Et Zucc [27]. However, there are few reports about the application of AMF agents in the artificial cultivation of *P. notoginseng*. Some studies have shown that there is abundant diversity and community structure of AMF in the rhizospheric soil of *P. notoginseng* and that *Glomus* is the dominant genus of AMF at different ages [28,29]; however, direct evidence of notoginsenoside accumulation in response to AMF is lacking. In our recent research, we reported that the contents of notoginsenoside were increased by AMF (*Glomus intraradices* and *G. etunicatum*) through the methyl jasmonate pathway [30]. As the colonization rate reaches a high level, AMF can significantly improve the uptake efficiency of plants in terms of phosphate, mineral elements and water contents, thereby increasing the biomass and contents of metabolites [31,32]. In addition, AMF participate in the regulation of metabolite biosynthesis by changing the concentrations of signal substances such as phytohormones and phenylpropanoids [33,34,35]. However, the effects of AMF on the contents and species of PNSs need to be elucidated. 

Metabolomics is a discipline related to the detection and exploration of the intervention-induced variation in metabolites in biological systems [36,37,38], in which liquid chromatography–mass spectrometry (LC–MS) based on metabolomic profiling in combination with statistical methods is currently used to determine whether there are bioactive substances in medicinal plants. Ultrahigh-performance liquid chromatography (UPLC)–electrospray ionization (ESI)–MS and tandem mass spectrometry (MS/MS) have been demonstrated to be highly sensitive and specific analytical methods for the analysis of ginsenosides, especially for screening and identifying trace and minor ginsenosides that display bioactivity [39,40,41,42].

In this study, we comprehensively analyzed the changes in *P. notoginseng* terpenoids in response to AMF via UPLC–ESI–MS/MS and elucidated the mechanism by which PNs accumulate. These findings shed new light on the scientization and rationalization of the use of AMF in the ecological planting of medicinal plants.

## 2. Results 

### 2.1. Terpenoid Profiling of P. notoginseng via UPLC–ESI–MS/MS 

**Figure 1:**
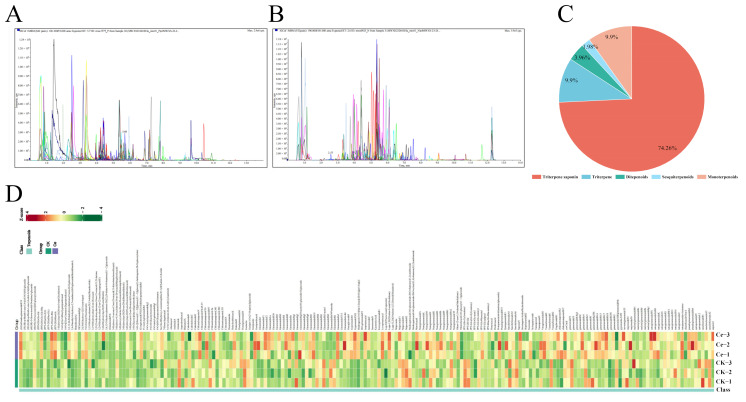
Qualitative and quantitative analyses of terpene metabolites in *P. notoginseng*. Multi-peak plots of multiple reactions monitoring metabolite detection in positive (**A**) and negative (**B**) ion modes. (**C**) Type and proportion of putative terpene metabolites. (**D**) Heatmap of terpene metabolites.

In this study, we focused on the changes in the composition and contents of *P. notoginsneg* terpenoids, especially triterpene glycosides, under the treatment of the AMF *Glomus etunicatum*. These terpenoids were quantitatively analyzed via multiple reaction monitoring modes, including positive ion mode (Figure 1A) and negative ion mode (Figure 1B). A total of 202 putative terpenoids were detected, including 150 triterpene glycosides, 20 nonglycosylated triterpenoids, 20 terpenes, eight diterpenoids and four sesquiterpenes (Figure 1C and Table S1). Triterpene glycosides were the main terpenoids, accounting for 74.26%, followed by other triterpenoid compounds (9.90%) and terpenes (9.90%) (Figure 1D), indicating that the terpenoid-dominated bioactive substances mainly originated from the triterpene glycosides in *P. notoginseng*.

### 2.2. Multivariate Statistical Analysis

**Figure 2:**
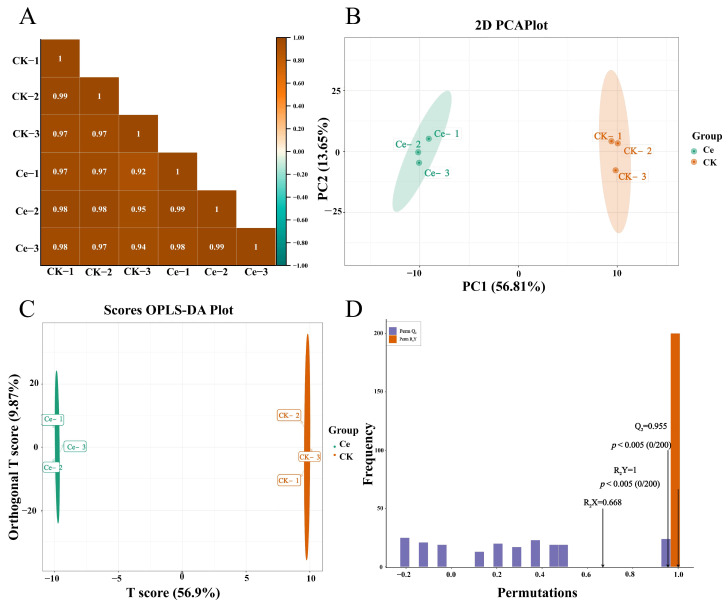
Analysis of the identified terpene metabolites in *P. notoginseng* roots under AMF treatment. (**A**) Pearson’s correlation coefficient between samples from the Ce and CK groups. (**B**) Principal component analysis (PCA) of the CK and Ce samples. (**C**) Orthogonal partial least squares discriminant analysis (OPLS–DA) model plot of the identified terpene metabolites in the Ce and CK groups. (**D**) OPLS–DA validation diagram.

Multivariate statistics were used to analyze the putative terpenoids in the Ce and CK groups. The results of Pearson’s correlation analysis revealed that there was a significant correlation (*p* < 0.05) among the biological replicates within each group or between groups Ce and CK (Figure 2A). The method of principal component analysis (PCA) is usually used to assess the overall differences among group samples and the magnitude of variation among samples within groups. As shown in the PCA plot, there was a significant difference between groups Ce and CK, and two principal components, PC1 and PC2, were extracted and accounted for 56.81% and 13.65% of the variability, respectively (Figure 2B). The three biological replicates of groups Ce and CK were concentrated in the left and right middle of the axis, respectively. The samples in the Ce and CK groups were directly divided into two groups and presented different accumulation patterns of these terpene metabolites, directly indicating that the inoculation of AMF in the roots of *P. notoginseng* could affect the synthesis and accumulation of triterpene glycosides. The orthogonal partial least squares discriminant analysis (OPLS–DA) mode is usually used to screen the identified metabolites and to evaluate the differentially abundant metabolites in metabolomics analysis. There was a clear separation between groups Ce and CK (Figure 2C), and a high predictability (Q2) and strong goodness of fit (R_2_X, R_2_Y) between both groups (Q2 = 0.955, R_2_X = 0.668 and R_2_Y = 1.000), which demonstrated that these modes were stable and reliable for identifying the differential terpenoid metabolites (Figure 2D). In brief, the above results indicated that there was a significant difference between the two terpene metabolic profiles of the Ce and CK groups.

### 2.3. Differential Terpene Metabolites between the AMF and CK Groups

**Figure 3:**
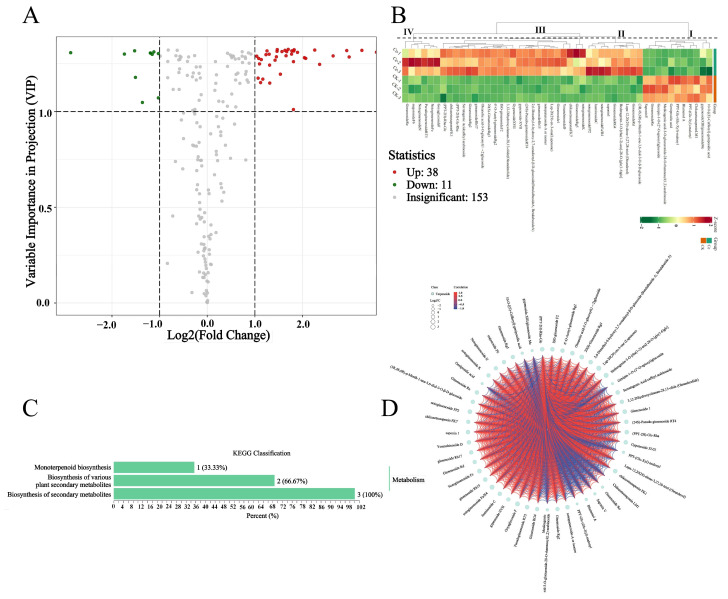
Differentially accumulated terpene metabolites between the Ce and CK groups. (**A**) Volcano plot of the differentially abundant metabolites. (**B**) Heatmap of the differentially abundant metabolites. (**C**) KEGG classification chart of differentially abundant metabolites. (**D**) Chord diagrams of differentially abundant metabolites used for correlation analysis. In (**A**), the red and green circles represent metabolites that are significantly upregulated and downregulated in group Ce, respectively, and the gray circles represent metabolites that are not significantly different between Ce and CK groups.

The differential terpene metabolites were screened using the criteria of a fold change ≥2 or ≤0.5 and variable importance in projection (VIP) ≥1. A total of 49 differential terpene metabolites were identified between groups Ce and CK, of which 38 terpenoids were upregulated, and 11 metabolites were downregulated in the Ce group compared with those in the CK group (Figure 3A,B and Table S2). Most of the upregulated differentially abundant metabolites were mainly triterpene glycosides, which can be categorized into three classes, and the largest class consisted of RNs (Figure 3B). The KEGG analysis results revealed that only three differential terpenoid metabolites were enriched in three pathways: the biosynthesis of secondary metabolites, the biosynthesis of various plant secondary metabolites and monoterpenoid biosynthesis (Figure 3C and Table S3). According to the annotation, the differential compounds enriched in the pathways of the biosynthesis of secondary metabolites and biosynthesis of various plant secondary metabolites are most likely those metabolites that are involved in ginsenoside biosynthesis (https://www.genome.jp/dbget-bin/www_bget?map00999, accessed on 15 June 2024; https://www.genome.jp/dbget-bin/www_bget?map01110, accessed on 15 June 2024). Importantly, there were significant positive correlations among the relative abundances of differential terpene metabolites (Figure 3D), suggesting that the accumulation of differentially abundant metabolites, particularly triterpene glycosides, may be triggered by the same factors, such as AMF.

### 2.4. Accumulation of PNSs Triggered by an AMF

**Figure 4:**
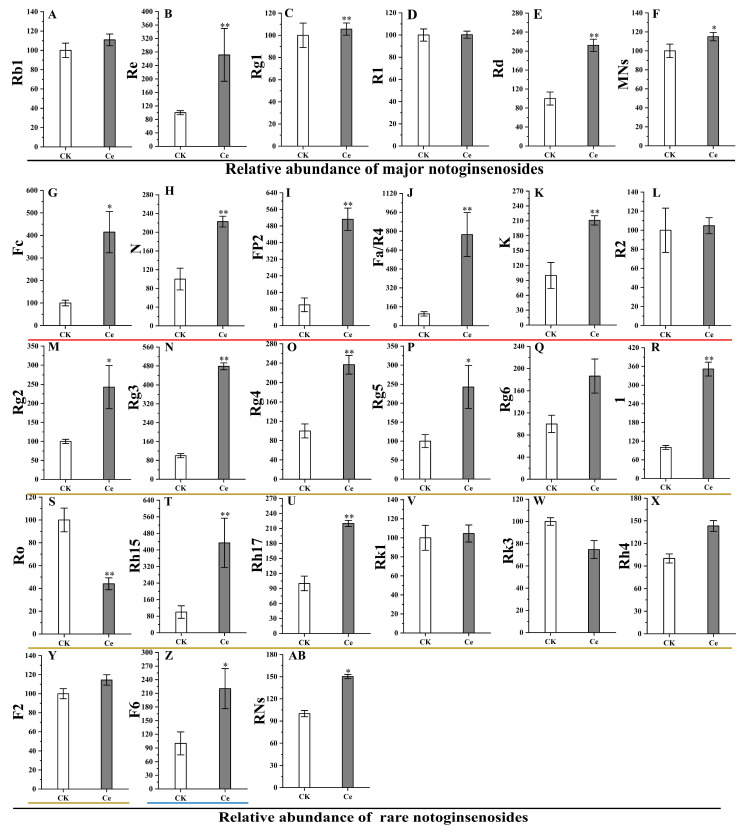
Effects of AMF on the relative abundances of major notoginsenosides (MNs) and rare notoginsenosides (RNs) in *P. notoginseng*. (**A**) Ginsenoside Rb1. (**B**) Ginsenoside Re. (**C**) Ginsenoside Rg1. (**D**) Notoginsenoside R1. (**E**) Ginsenoside Rd. (**F**) Total MNs. (**G**) Notoginsenoside Fc. (**H**) Notoginsenoside N. (**I**) Notoginsenoside FP2. (**J**) Notoginsenoside Fa/R4. (**K**) Notoginsenoside K. (**L**) Notoginsenoside R2. (**M**) Ginsenoside Rg2. (**N**) Ginsenoside Rg3. (**O**) Ginsenoside Rg4. (**P**) Ginsenoside Rg5. (**Q**) Ginsenoside Rg6. (**R**) Ginsenoside 1. (**S**) Ginsenoside Ro. (**T**) Ginsenoside Rh15. (**U**) Ginsenoside Rh17. (**V**) Ginsenoside Rk1. (**W**) Ginsenoside Rk3. (**X**) Ginsenoside Rh4. (**Y**) Ginsenoside F2. (**Z**) majoroside F6. (**AB**) Total RNs. * and ** indicate a significant difference in the relative abundance between groups Ce and CK at *p* < 0.05 and *p* < 0.01, respectively.

**Figure 5:**
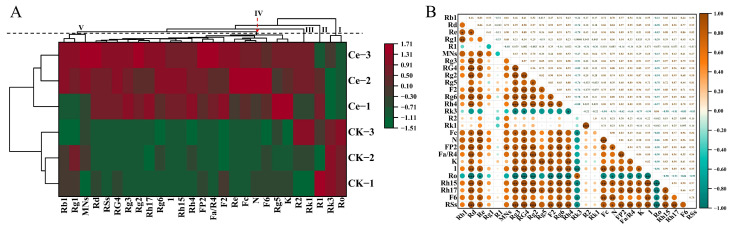
Cluster analysis of five major notoginsenosides (MNs) and 17 rare notoginsenosides (RNs) of *P. notoginseng* (**A**) and Pearson’s correlation analysis among these saponins (**B**). As shown in (**A**), the cluster analysis was conducted with parameters “Manhattan distance 4” in the tool and “heatmap with dendrogram” provided by the software Origin 2021. In (**B**), the circles’ earthy yellow and blue–green colors indicate positive and negative correlations between two indicators, which correspond to the horizontal and vertical coordinates, respectively; the darker the circle is, the greater the correlation between the two indicators. *, ** and *** denote significant correlation at the levels of *p* < 0.05, *p* < 0.01 and *p* < 0.001, respectively.

The effects of AMF on the accumulation of PNSs were determined by determining the relative abundances of five major and 20 rare notoginsenosides, which are the main pharmacologically active substances. As shown in Figure 4, there was a significant increase in the relative levels of MNs and RNs. The relative abundances of two of the five MNs, ginsenosides Rd and Re, significantly increased by 2.11- and 2.90-fold, respectively, resulting in a significant increase in the total number of MNs. In the list of 20 common *P. notoginseng* RNs, the relative abundances of five unique notoginsenosides, seven ginsenosides and one majoroside significantly increased, but those of five ginsenosides did not significantly differ. Among the five unique notoginsenosides, the relative abundance of notoginsenoside Fa increased the most, by 7.88-fold, followed by notoginsenosides FP2 (5.13-fold), Fc (4.19-fold), N (2.24-fold) and K (2.10-fold). The increase of ginsenosides Rg3 (4.78-fold) was the greatest among the RNs, followed by ginsenoside Rh15 (4.11-fold), ginsenoside 1 (3.52-fold), ginsenoside Rg5 (2.48-fold), ginsenoside Rg2 (2.46-fold), ginsenoside Rg4 (2.35-fold) and ginsenoside Rh17 (2.20-fold). The majoroside F6 was upregulated 2.17-fold. As a result, the total number of RNs in group Ce was significantly different from that in the CK group. In addition, the results of the cluster analysis revealed that the five major and 17 rare notoginsenosides could be divided into five branches at a distance value of four, in which ginsenosides Rk3 and Ro were categorized as branch Ⅰ; notoginsenoside R1 and both ginsenoside Rk1 and notoginsenoside R2 belonged to branches Ⅱ and Ⅲ, respectively; 18 RNs and two MNs, including notoginsenosides K, N, Fc and Fa/R4, and ginsenosides Rg5, Re, F2, Rh15, 1, Rh4, Rh17, Rg6, Rg2, Rg3, Rg4 and Rd, majoroside F6 and total RSs, were clustered into branch Ⅳ; ginsenosides Rg1 and Rb1, and the total MNs were grouped into branch Ⅴ (Figure 5A). Interestingly, the differential notoginsenosides were more easily clustered into one branch, especially the RNs (Figure 5A). The Pearson correlation results confirmed that there were high correlation coefficients among RNs, e.g., between notoginsenosides N and Fc or between notoginsenoside Fa/R4 and ginsenoside Rg2 (Figure 5B). It is speculated that the biosynthesis and accumulation of some notoginsenosides share the same pathways and are triggered by similar factors, such as AMF.

## 3. Discussion

**Figure 6:**
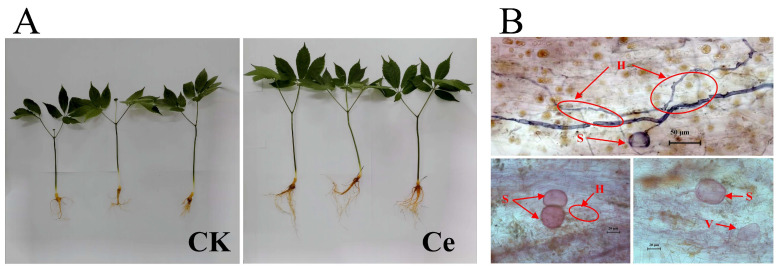
The effects of AMF agent on the growth of *P. notoginseng* (**A**) and the typical morphological microstructure of AMF in symbiotic mycorrhizae (**B**). H, hyphae; S, spores; V, vesicles.

As an important medicinal material in China, notoginseng radix et rhizoma holds the first place in the sale volume of the whole patent medicine market, and the market size of a single species has exceeded 10 billion yuan [5]. Given the increasing demand for yield and quality standards, it is particularly important to cultivate *P. notoginseng* with high notoginsenoside contents by biological means that are widely used in ecological planting. Increasing evidence has shown that AMF not only contributes to the growth and development of host plants but also plays a positive role in improving the production and accumulation of bioactive compounds in medicinal plants, e.g., *A. annua*, *Polygonatum kingianum* Collett & Hemsl., *Salvia officinalis* L., *S. miltiorrhiza* Bge., *Melissa officinalis* L., *Passiflora alata* Curtis., *Ocimum basilicum* L. and *Thymus vulgaris* L. [23,43,44,45,46,47,48,49]. In this study, it was found that the application of the AMF *G. etunicatum* could significantly promote the growth of *P. notoginseng* and lead to the formation of symbiotic mycorrhiza mycorrhizae with the typical morphological microstructure of AMF, including hyphae, spores and vesicles (Figure 6). However, there are few reports about the changes in the constituents of PNSs, especially RNs, triggered by AMFs.

In this study, UPLC–tandem mass spectrometry (UPLC–MS/MS), which is widely used for targeting, was used to analyze the changes in terpenoids. A total of 150 triterpene glycosides in *P. notoginseng* were identified, accounting for 74.26% of the terpenoids (Figure 1 and Table S1). The number of types of triterpene glycosides was significantly greater than that of 62 in *P. ginseng* and seven in *Eleutherococcus senticosus* (Rupr. & Maxim.) [50,51] but significantly lower than that of 361 in *P. ginseng* roots that were planted in different environments, reported by Sun et al. [52]. The results of multivariate statistics revealed that the metabolomics data of the Ce and CK groups were very reliable and logical (Figure 2), which satisfied our need for further analysis.

Regarding the change in differentially abundant metabolites, the number of upregulated PNSs was greater than that of downregulated PNSs, with a ratio of 38:11, and the upregulated PNSs were mainly MNs and RNs, such as ginsenosides Rd, Re, Rg2, Rg3, Rg4, Rg5, Rh15 and Rh17, and notoginsenosides Fa, FP2, Fc, N and K (Figure 3 and Figure 4). The accumulation of differentially abundant metabolites, particularly triterpene glycosides, may be triggered by AMF. Previous studies confirmed that the inoculation of AMF significantly increased the biosynthesis and accumulation of secondary metabolites, e.g., artemisinin [23,24], glycyrrhizin and liquiritin [25,26], aloe–emodin [29] and phenolic acids [53].

In this study, we focused on the change in the relative abundance of terpenoids, especially triterpene glycosides, in *P. notoginseng* taproots under AMF treatment. The results revealed that the relative abundance of MNs, ginsenosides Rd and Re and seven RNs significantly increased, leading to a significant increase in total PNSs. These results are similar to those of Dai et al. [30] and Cao et al. [12], who reported that the inoculation of a mixture of AMF (*G. intraradices* and *G. etunicatum*) or single *G. intraradices* could promote the growth and MN (notoginsenoside R1 and ginsenosides Re, Rb1 and Rd) accumulation of *P. notoginseng* by increasing the activities of key enzymes and the expression levels of encoding genes. The secretion of compounds that trigger plant signaling systems by AMF is the main explanation for the accumulation of secondary metabolites [33]. In addition, the growth promotion effect of AMF through changes in the mineral composition of rhizosphere soil, such as improvements in the availability of phosphates, is also one of the main ways in which AMF increases the accumulation of plant secondary metabolites [54,55].

A low concentration does not indicate low pharmacological action. Increasing evidence has confirmed that many RNs have strong pharmacological activity and play important roles in the treatment of cardiovascular and cerebrovascular diseases, e.g., ginsenoside Rg3 can reduce cardiac toxicity and inhibit mast cell-mediated allergies [14,56,57], and ginsenoside Rh4 has strong anticancer and anti-inflammatory effects [58,59,60]. Therefore, increasing the contents of RNs has become a research hotspot. Biological transformation and steam processes are common methods that are also widely used in the acquisition of valuable metabolites [61,62,63]. A study revealed that *Aspergillus fumigatus* can transform PNSs into 14 products that were isolated and identified as ginsenosides C–K, 20 (R/S)–Rg3, Rg5, 20 (R)–Rh1, Rk1, Rh4, Rk3, 20(S)-protopanaxatriol, 20(S)–I, 20 (R/S)–Rg2, and notoginsenosides 20 (R/S)–R2, respectively [64]. Dong et al. [65] reported that the contents of ginsenosides Rg1, Re, Rb1, Rd and Rg5 and notoginsenoside R1 increased significantly after the steaming process. The greatest advantage of our work is that the increase in RNs appeared during the growth of *P. notoginseng*, rather than during transformation from other saponins after harvest, avoiding the uncertainty of transformation and the internal transformation from one saponin to the other.

In addition, the differentially expressed notoginsenosides were more easily clustered into one branch and presented a high positive correlation, especially RNs (Figure 5). These notoginsenosides may share the same pathways and be triggered by similar factors, such as AMF. A similar hypothesis has also been mentioned by many researchers [66,67,68,69].

## 4. Materials and Methods

### 4.1. Plant Materials and Pot Experiments

The annual *P. notoginseng* seedlings used for the treatments were collected from the Zhuang–Miao Autonomous Prefecture of Wenshan Yunnan Province, which has good growth conditions and similar biomass. AMF strains: Spores of *G. etunicatum* mixed in the soil were purchased from the Institute of Root Biology, Wuhan Yangtze University. After morphological identification, the soil containing *G. etunicatum* spores was mixed into the sterilized soil substrate at a ratio of 1:20, and then the seeds of maize and clover were planted into the soil, in which the root systems were used for spore propagation under semiclosed conditions. There were two groups: the AMF treatment group (Ce) and the blank control group (CK) in this experiment [30].

The cultivation substrate used in this experiment was composed of nutrient soil and vermiculite (18:1), which were mixed evenly and sterilized with intermittent humid heat (121 °C, 2 h; 2 times). After drying, the sterilized cultivation substrate was subpackaged into clean pots, and each pot contained 1.5 kg of mixed soil. Before planting, the *P. notoginseng* seedlings described above were rinsed with purified water to remove the attached soil and sprayed with benomyl to inhibit indigenous strains. Three *P. notoginseng* plants were planted in each pot, and 20 g of AMF soil containing approximately 100 *G. etunicatum* spores was placed around the roots. There were eight pots in each group, with a total of 24 plants. *P. notoginseng* has strict requirements for environmental conditions. The seedlings were planted in a greenhouse at 25 ± 3 °C and 60 ± 5% relative humidity, avoiding direct sunlight and water accumulation and maintaining ventilation. After six months, the fresh roots were harvested and used for widely targeted metabolomic analysis. The remaining samples were stored at −80 °C [12,30].

### 4.2. Sample Preparation and Extraction 

The freeze-dried samples of each group were crushed via a mixer mill (MM 400, Retsch, Haan, German) for 90 s at 30 Hz and were mixed thoroughly. A total of 50 mg powder was weighed and extracted with 1200 μL of 70% aqueous methanol prechilled to −20 °C containing internal standards. The extraction was carried out by vortexing every 30 min for 6 times, and each time was 30 s. Centrifugation was set up at 12,000 rpm for 3 min, and the supernatants were filtered with a 0.22 μm filter membrane. The filtrate was stored in a sample vial for UPLC–ESI–MS/MS (SCIEX, Shanghai, China) analysis.

### 4.3. UPLC Conditions

Terpene component analysis was performed via a UPLC–ESI–MS/MS system (UPLC, ExionLC™ AD, SCIEX, Shanghai, China, https://sciex.com.cn/, accessed on 27 March 2024) and a tandem mass spectrometry system (https://sciex.com.cn/, accessed on 27 March 2024). The analytical conditions were as follows: UPLC: column, Agilent SB–C18 (Agilent Technologies, Santa Clara, CA, USA, 1.8 µm, 2.1 mm × 100 mm); The mobile phase consisted of solvent A, pure water with 0.1% formic acid, and solvent B, acetonitrile with 0.1% formic acid. Sample measurements were performed with a gradient program that employed the starting conditions of 95% A and 5% B. Within 9 min, a linear gradient of 5% A and 95% B was programmed, and the composition of the gradient was maintained for 1 min. The flow velocity was set as 0.35 mL/min. The column oven was set at 40 °C, and the injection volume was 2 μL. The effluent was alternatively connected to an ESI–triple-quadrupole–linear ion trap (QTRAP)–MS (SCIEX, Shanghai, China) [70].

### 4.4. ESI–Q TRAP–MS/MS 

Linear ion trap (LIT) and triple quadrupole (QQQ) scans were acquired on a triple-quadrupole–linear ion trap mass spectrometer (Q–TRAP–linear ion trap mass spectrometer) and the AB4500 Q–TRAP–UPLC/MS/MS system equipped with an ESI Turbo Ion–Spray interface, operating in positive and negative ion modes and controlled by Analyst 1.6.3 software (AB Sciex, Concord, ON, Canada) [71]. The ESI source operation parameters were as follows: the source temperature and ion spray voltage (IS) were set at 550 °C and 5500 V (positive ion mode)/−4500 V (negative ion mode), respectively; ion source gas I (GSI), gas II (GSII) and curtain gas (CUR) were set at 50, 60 and 25 psi, respectively; and the collision-activated dissociation (CAD) was high. QQQ scans were acquired as multiple reaction monitoring (MRM) experiments with the collision gas (nitrogen) set to medium. Declustering potential (DP) and collision energy (CE) for individual MRM transitions were performed with further DP and CE optimization. A specific set of MRM transitions was monitored for each period according to the metabolites eluted within this period [72].

### 4.5. Qualitative and Quantitative Analyses of Terpene Metabolites 

The identification and structural analyses of the primary and secondary spectral data were conducted by mass spectrometry (MS) based on the MWDB database (Metware Biotechnology Co., Ltd. Wuhan, China) and some public databases, e.g., MassBank (http://www.massbank.jp/, accessed on 27 March 2024), KNAPSAcK (http://kanaya.naist.jp/KNApSAcK/, accessed on 29 March 2024), HMDB (http://www.hmdb.ca/, accessed on 29 March 2024) and PubChem (https://pubchemblog.ncbi.nlm.nih.gov/, accessed on 2 April 2024). Metabolomics data were processed using Analyst (Version 1.6.3, Sciex, Framingham, MA, USA) [71].

Metabolite quantification was performed using the MRM mode of QQQ MS. In the MRM mode, the quadrupole filters the precursor ions of the target substance and excludes the ions corresponding to other molecular weights to eliminate interference. Based on the MS data, the peak area integration was performed using MultiQuant version 3.0.2 (AB SCIEX, Concord, ON, Canada), and then the relative abundance was calculated [73].

### 4.6. Principal Component Analysis

The data used for unsupervised PCA should be unit-variance scaled first, and then the variabilities between and within groups were performed by statistics function prcomp within R (www.r-project.org, accessed on 1 May 2024). 

### 4.7. Hierarchical Cluster Analysis and Pearson Correlation Coefficients

The hierarchical cluster analysis (HCA) results of the metabolites were presented as heatmaps with dendrograms, while the Pearson correlation coefficients (PCC) between samples were calculated and drawn by the cor function in R (www.r-project.org, accessed on 1 May 2024). Both HCA and PCC were carried out by the R package Complex Heatmap (R 4.3.2). For HCA, normalized signal intensities of metabolites (unit-variance scaling) are visualized as a color spectrum [71]. 

### 4.8. Differentially Abundant Metabolite Selection and KEGG Annotation and Enrichment Analysis

Orthogonal partial least squares discriminant analysis (OPLS–DA) was employed to identify differentially abundant metabolites, and the parameters were set as VIP (VIP > 1) and absolute Log_2_^FC^ (|Log_2_^FC^| ≥ 1.0). The VIP values originated from the OPLS–DA results, which also contained score plots and permutation plots that were generated via the R package of MetaboAnalystR (https://github.com/xia-lab/MetaboAnalystR, accessed on 1 May 2024). The data were normalized by log transform (log_2_) and mean centering before OPLS–DA. A permutation test (200 permutations) was performed so as to avoid overfitting.

The identified metabolites were annotated via the KEGG compound database (http://www.kegg.jp/kegg/compound/, accessed on 27 March 2024) and then were mapped to the KEGG pathway database (http://www.kegg.jp/kegg/pathway.html, accessed on 27 March 2024). Pathways with significantly regulated metabolites were then subjected to metabolite set enrichment analysis (MSEA), and their significance was determined via hypergeometric test *p*-values. 

### 4.9. Data Analysis

Significant differences were tested with an unpaired *t*-test at the 0.05 level. Origin 2021 and R (www.r-project.org, accessed on 27 March 2024) were used for the data visualization. Since the samples in each group were randomly divided into three parallel samples in powder form for UPLC–ESI–MS/MS analysis, the degree of freedom (*n*) used for the data analysis was three (*n* = 3). The standard deviation (SD) was used for error bars, *n* ≥ 3. 

## 5. Conclusions 

In this study, UPLC–MS/MS widely targeted metabolomics was used to analyze the relative abundance difference in the terpenoids in notoginseng Radix et rhizoma between the Ce and CK groups. A total of 202 putative terpenoid metabolites were detected, of which 150 triterpene glycosides were identified, accounting for 74.26% of the total. In total, 49 differentially abundant metabolites were identified between the Ce and CK groups, of which 38 terpene compounds were upregulated, and 11 metabolites were downregulated, respectively, and most of the upregulated differentially abundant metabolites were triterpene glycosides. The results showed that the relative abundances of two MNs and 13 RNs, including majoroside F6, ginsenosides 1, Rd, Re, Rg2, Rg3, Rg4, Rg5, Rh15 and Rh17 and notoginsenosides Fa, FP2, Fc, N and K significantly increased, and these saponins, especially RNs, were more easily clustered into one branch and had a high positive correlation. It is speculated that the biosynthesis and accumulation of some RNs share the same pathways as those triggered by AMF. This study provides a new way to obtain more notoginsenoside resources, particularly RNs, and would be a reference for implementing ecological planting of *P. notoginseng*.

## Data Availability

Data are contained within the article and Supplementary Materials.

## Supplementary Materials

The following supporting information can be downloaded at: https://www.mdpi.com/article/10.3390/molecules29133235/s1. The Supplementary Materials are available online. Table S1. List and characteristics of the terpene metabolites identified and quantified in *P. notoginseng*. Table S2. List of differentially accumulated terpene metabolites in the Ce vs. CK dataset. Table S3. List of KEGG pathways in the Ce vs. CK dataset.
